# Adaptive Localization-Free Secure Routing Protocol for Underwater Sensor Networks

**DOI:** 10.3390/s26010017

**Published:** 2025-12-19

**Authors:** Ayman Alharbi, Saleh Ibrahim

**Affiliations:** 1 Department of Computer Engineering and Networks, Umm Al-Qura University, Makkah 24382, Saudi Arabia; 2 Department of Electrical Engineering, Taif University, Taif 21974, Saudi Arabia; saleh@eng.cu.edu.eg; 3 Department of Computer Engineering, Cairo University, Cairo 12613, Egypt

**Keywords:** underwater acoustic networks, depth-based routing, secure routing

## Abstract

Depth-based probabilistic routing (DPR) is an efficient underwater acoustic network (UAN) routing protocol which resists the depth-spoofing attack. DPR’s optimal value of the unqualified forwarding probability depends on the UAN topology, condition, and threat state, which are highly dynamic. If the static forwarding probability used in DPR is set too low for the current state, packet delivery ratio (PDR) drops. If it is set too high, unnecessary forwarding occurs when the network is not under attack, thus wasting valuable energy. In this paper, we propose a novel routing protocol, which uses a feedback mechanism that allows the sink to continuously adapt the unqualified forwarding probability according to the current network state. The protocol aims to achieve an application-controlled desired delivery ratio using one of three proposed update algorithms developed in this work. We analyze the performance of the proposed algorithms through simulation. Results demonstrate that the proposed adaptive routing protocol achieves resilience to depth-spoofing attacks by successfully delivering more than 80% of generated packets in more than 95% of simulated networks, while avoiding unnecessary unqualified forwarding in normal conditions.

## 1. Introduction

The harsh underwater environment takes its toll on the performance of underwater communication networks. The highly dynamic nature of the communication channel, the relatively low channel capacity, the extremely long propagation delays, and the limited energy availability affect the design of all layers of underwater networks [1]. Underwater sensor networks routing protocols are designed to cope with intermittent connectivity, frequent topology changes, and the lack of precise node positioning. Consequently, the design of efficient routing strategies has become a central research focus, serving as the foundation for higher-level performance improvements in underwater communication systems [2].

Depth-based routing (DBR) [3] is a very successful class of underwater routing protocols due to its energy efficiency in the absence of full node localization. DBR opportunistically utilizes relay nodes to deliver packets from source nodes, which are typically at deeper levels, to the sink nodes, which are typically at the surface level. Due to its efficiency and low cost, DBR has attracted a lot of interest and is the basis of plenty of research effort aiming to improve DBR performance.

Unfortunately, a particular vulnerability known as the depth-spoofing attack, described in [4], allows adversaries to severely hinder the ability of DBR protocols to deliver packets. In this attack, a malicious node exploits the opportunistic nature of DBR by claiming to be close to the surface, thus inhibiting nearby relay nodes from participating in routing. A few mechanisms for resisting the depth-spoofing attack can be found in the literature. In RPR [5], the depth-spoofing attack is mitigated by using a forwarding-node selection technique instead of purely opportunistic routing. However, forwarding-node selection incurs high control overhead to keep up with highly dynamic underwater network topologies. In [6], the author utilized shared-key signed packet headers to secure depth information to protect against depth-spoofing by adversaries. However, the shared-key pre-distribution limits scalability.

The depth-based probabilistic routing protocol (DPR), recently proposed in [4], tackles the depth-spoofing attack by encouraging suboptimal relay nodes to randomly forward packets, thus circumventing any potential attempts by a malicious neighbor to deter packet delivery. By increasing the probability of unqualified forwarding, denoted p, DPR achieves high packet delivery ratios (PDRs) in the presence of a depth-spoofing attacker, comparable to the delivery ratios under normal operation, thus mitigating the effect of this attack.

However, as the unqualified forwarding probability increases, energy consumption proportionally increases even when the network is not under attack. In lightly loaded networks, DPR can incur up to a 30% energy overhead compared to DBR when the network is not under attack. Therefore, careful adjustment of the forwarding probability is crucial for optimizing the tradeoff between delivery ratio and energy efficiency. When the network state is dynamic, the existing DPR static adjustment methodology of the unqualified forwarding probability fails to achieve the desirable delivery ratio or maintain energy efficiency. An unqualified forwarding probability that is optimal at a certain traffic load may not be optimal for another. It is worth noting that simulation results of DPR show that denser networks require a smaller p than that required by more sparse networks to achieve the same level of depth-spoofing resistance. Moreover, it is desirable to activate the unqualified probabilistic forwarding mechanism only when the network is under attack and disable it otherwise.

Periodically adjusting the forwarding probability to cope with the dynamic status of the network can mitigate this issue. This requires estimating the status of the network and adjusting the forwarding probability accordingly. We refer to the time it takes the network to collectively respond to a change in network status as the convergence time. Fast convergence is crucial for ensuring timely and reliable data delivery, especially in dynamic environments, where network topology may change frequently due to node failures, mobility, or environmental conditions.

In this paper, we propose an adaptive probabilistic forwarding mechanism, in which the sink node measures the delivery ratio and broadcasts a periodic update message to notify all nodes to update their individual forwarding probability correspondingly. We develop three algorithms for updating forwarding probability and evaluating their performance in comparison to one another and to existing protocols.

The proposed Adaptive Localization-free Secure Routing (ALSR) protocol introduces three new features: (1) detection of depth-spoofing attacks and identification of affected sources by monitoring delivery ratio of each source node, (2) mitigation of depth-spoofing attacks using novel energy-efficient algorithms for dynamically adjusting the forwarding probability, p, for each relay node based on the network density to achieve the desirable delivery ratio, and (3) investigate the convergence time of various probability update algorithms at the onset of a depth-spoofing attack.

## 2. Background and Related Work

In this study, we focus on localization-free underwater routing protocols and their secure extensions. Localization-free routing protocols depend on depth information, which avoids full localization. Accurate one-dimensional depth information is readily available through simple pressure sensors, whereas accurate underwater localization uses telemetry, which is a resource-intensive task due to acoustic channel characteristics, long propagation delays, and limited bandwidth.

From a security standpoint, depth-based routing protocols introduce a well-known vulnerability known as depth-spoofing (sinkhole) attacks, which directly exploit falsified depth advertisements. Classical depth-based routing protocol, DBR, and other energy-efficient variants such as EEDBR [7], LDBR [8], EECOR [9], and related protocols inherit the same fundamental reliance on advertised depth information for routing eligibility. Therefore, they share vulnerability to depth-spoofing attacks. As a result, the proposed ALSR anti-depth-spoofing mechanism is applicable to all such depth-based routing protocols to enhance their resilience while preserving their original energy-optimization objectives.

Classical DBR protocol and its recently improved versions are discussed in this section, emphasizing the key differences that set our approach apart from existing versions. In addition, we show the importance of curve-fitting methodology in our proposed model.

### 2.1. Depth-Based Routing (DBR) Protocol

The initial and fundamental concept of the DBR protocol was to circumvent the dependency on complex localization information for each node in the network and instead rely solely on depth information to design a lightweight routing algorithm [3]. Besides its localization-free advantage, DBR requires minimal control overhead. DBR does not rely on periodic neighbor discovery, route setup, or explicit coordination messages. Instead, nodes implicitly coordinate by advertising their depth values within the headers of forwarded data packets, making DBR particularly suitable for dynamic and energy-constrained underwater environments.

The DBR algorithm determines forwarding eligibility based on the depth of each receiving node. A node with a lower depth has higher priority than its neighbors to forward received packets, while packets from deeper nodes are discarded. Each eligible node applies a holding time calculation, ensuring that the node closest to the surface is prioritized to forward before its neighbors. The holding time for a potential forwarder can be calculated as follows:

(1) $$T_{H}=\frac{2R}{V}\cdot \frac{\left(R-\Delta d\right)}{\delta }$$

where V is the acoustic signal propagation speed, R is the node communication range, $\Delta d=d_{sender}-d_{forwarder}$ is the hop depth gain, and δ ∈(0 ,R] is a constant that determines the maximum holding time. Using this simplified methodology, each node waits a holding time then forwards the held packet only if it hears no neighbor closer to the surface forwarding the same packet.

### 2.2. Enhanced Depth-Based Routing Protocols

The advancement in DBR has attracted numerous researchers to enhance its performance for different metrices. To ensure energy-efficient routing based on depth-routing strategies, EEDBR [7], LDBR [8], and EECOR [9] enhance the forwarding eligibility process by considering residual energy and other optimization factors. Upon making a forwarding decision, EEDBR and LDBR attempt to extend the overall network lifetime by considering remaining energy levels in addition to depth feature. Unlike in DBR protocol, the sender in EEDBR protocol selects candidate forwarders by ensuring two conditions: each chosen neighbor must have a smaller depth than the sender and sufficient residual energy so that forwarding tasks are distributed more evenly. By combining depth with residual energy as selection criteria, this enhancement leads to balanced energy consumption across the network and avoids routing holes. To ensure better selection of eligible forwarders, DCEER [10] utilizes data fusion strategies to eliminate any potential duplicated transmission. Nodes with higher residual energy will be selected over lower residual energy ones based on genetic algorithms and an enhanced cluster head selection method. Closely related to this approach, the authors in [11] introduced the Energy-Efficient Clustering Protocol EECP, which was proposed to address energy conservation and network lifetime. The network is divided into depth-based horizontal layers. Within each layer, a cluster head (CH) is selected primarily on residual energy. The improved version, EECP-NN, refines this by also considering the distance to the nearest neighbor/sink as a secondary metric for CH election. Results show that the integration of depth-based clustering with adaptive CH selection maximizes energy conservation, balances node load, and extends the overall lifetime of underwater wireless sensor networks. Depth-based routing has also been utilized along with edge-assisted election protocol to minimize energy consumption and prolong network lifetime. The Edge-Assisted Depth-Based Stable Election Protocol EA-DBSEP-IoUT [12] integrates residual energy, depth, and connectivity factors for intelligent cluster head election, while leveraging reinforcement learning and edge-assisted data aggregation to minimize redundant transmissions.

Since underwater wireless networks are highly vulnerable to the harsh marine environment and are constrained by limited energy resources, adaptive mechanisms have emerged as some of the most effective solutions to overcome these challenges. In [13], the authors introduced the SEEC (CTPSEEC) algorithm, which uses adaptive techniques to control node transmissions. This helped to maintain acceptable power levels and minimize unnecessary energy consumption. In addition to that, SEEC utilizes a cluster-based protocol to split sparse areas into equal sub-areas and adjust sink locations to enhance power consumption. In similar work, the authors in [14,15] incorporated adaptive power mechanisms to control forwarding decisions. Each node keeps an up-to-date neighbor table with information about depth and residual energy levels to be used for selecting next-hop relay nodes. The study in [14] focused on integrating residual energy awareness with hole-avoidance strategies to maintain efficient routing under dynamic underwater conditions. In contrast, the more recent work in [15] advanced this approach by introducing a dual-weight adjustment mechanism with cross-layer design, incorporating both local link quality (via received signal strength) and global stability (through a Link Energy Stability Factor), which further enhanced energy efficiency and prolonged network lifetime by nearly 39% compared with EEDBR [7]. Thus, while both works emphasized adaptive power mechanisms, the second contribution extended the benefits by combining energy-aware forwarding with global stability considerations. Overall, adaptive power control was shown to significantly reduce redundant transmissions and balance energy usage across nodes, thereby extending the network lifetime and improving delivery reliability.

### 2.3. Depth-Based Routing Protocol Security

It has been shown that depth-based routing schemes are vulnerable to the security threat known as the depth-spoofing attack. In this attack, a malicious node is placed within the transmission range of a source node. The malicious node then advertises a falsified depth value to mislead neighboring nodes into relying on this malicious node as the preferred forwarder. As a result, all packets forwarded by a malicious node may be dropped, delayed, or misrouted, ultimately preventing them from reaching the sink and severely degrading the overall network performance. Several countermeasure strategies have been proposed to secure the depth-spoofing (sinkhole) attack. For example, the resilient pressure-based routing (RPR) protocol prevents depth-spoofing by combining cryptography techniques with routing algorithms. Each node has a certified key pair to sign its packets so that only legitimate nodes can process them. Upon forwarding a packet, the sender assigns two depth thresholds for selecting a window of neighbors that are allowed to participate in forwarding the packet. Payloads are also protected with the gateway’s public key, preventing unauthorized modifications and impersonation attempts. In addition to that, the fixed depth-gain threshold used in forwarding decisions of a packet as in DBR is enhanced by the sliding window mechanism having both lower and upper bound threshold values in each packet’s header. Only the nodes that have depth in this threshold window are allowed to forward the packet. Although this randomization process, along with encryption, can prevent depth-spoof attacks from succeeding for a high percentage of time, RPR suffers high transmission costs since the use of randomized threshold windows generally results in path formations with more hops relative to the opportunistic DBR approach. Moreover, key management processes are considered very challenging in the underwater environment due to several factors such as water currents, temperature, and salinity, which affect communication, causing packet loss, signal attenuation, and even the loss of cryptographic keys [16]. In [6], the authors proposed a methodology based on encrypting only the packet header of DBR packet rather than the entire payload. This selective encryption strategy reduces the computational and energy overhead associated with decrypting full data packets. While this approach is effective in mitigating depth-spoofing attacks and securing routing decisions, it depends on pre-distributed cryptographic keys which limit the scalability of the network. It also suffers from a weakness: compromising a single node could expose the keys to potential intruders.

### 2.4. Depth-Based Probabilistic Routing (DPR)

The DPR protocol [4] mitigates the effect of depth-spoofing by utilizing probabilistic methods to handle duplicate packets. Unlike traditional forwarding/dropping decisions in DBR and RPR, DPR keeps received packets within candidate forwarders to decide—based on a set probability—whether to forward or drop them when the timer expires. This adjustment ensures that even when a malicious node attempts to spoof its depth so that all other candidate forwarders drop received packets, a fraction of them are still successfully delivered. By carefully tuning the forwarding probability according to network density, DPR maintains strong resistance to spoofing while keeping energy overhead under control. Although DPR effectively resists depth-spoofing attacks by introducing probabilistic forwarding, its main shortcoming lies in the dependency of a static unconditional forwarding probability. The optimal probability requires careful adaptation on the network’s topology, traffic load, and whether an attack is taking place—factors that are inherently dynamic in underwater acoustic networks. If the probability is set too low, DPR fails to deliver enough packets under attack, weakening its resilience. Conversely, if it is set too high, the protocol triggers redundant retransmissions even when no attack is present, leading to wasted energy and unnecessary overhead.

Table 1 provides a summary of state-of-the-art secure localization-free depth-based routing protocols, their security properties, and their key limitations.

## 3. Adaptive Localization-Free Secure Routing Protocol (ALSR)

### 3.1. Assumptions and Definitions

We assume that the network is centrally controlled by a sink node, which is collecting data from underwater nodes. The underwater nodes cooperatively participate in the relaying of data packets generated by a subset of nodes called the source nodes, S. We assume that nodes can authenticate control traffic originating from the sink node. We assume that the sink node knows the minimum desired data flow rate from each source node and can use this information to identify nodes that are suffering from connectivity problems. We assume that the header of each data packet will contain an authenticated source-generated sequence number. The sink node can estimate whether the flow from a certain source is affected by a black hole or a gray hole attack, based on the delivery ratio calculated by counting the number of missing sequence numbers from this source.

We define a security parameter called the desired delivery ratio, denoted K∈[0,1], which determines the acceptable per-source delivery ratio when the network is under a black hole or a gray hole attack.

### 3.2. ALSR Protocol Description

The main idea of ALSR is to dynamically circumvent sinkhole attacks. While relay nodes cannot directly detect the presence of a malicious node performing depth-spoofing attack, the sink node can detect the drop in packet delivery ratio (PDR) when such an attack is active. When the PDR is unsatisfactory, the sink node assumes there is an active sinkhole and adjusts the unqualified forwarding probability accordingly to increase the chances of delivery of packets affected by the sinkhole.

The proposed routing protocol classifies packets according to their source, s, and assigns to each packet a forwarding probability, $p_{s}$, corresponding to the packet source. By employing this classification, the sink can respond to attacks which affect the packets originating from a subset of sources without unnecessarily increasing all network traffic. Only those packets affected by the depth-spoofing attack will be given a higher forwarding probability. The optimal forwarding probability, $\hat{p_{s}}$, for packets originating from the source node, s, is affected by the network density and the threat level, $t_{s}$, affecting the packet stream from s. The protocol is designed with the goal of automatically finding and enforcing the optimal forwarding probability. This is achieved through an iterative update process.

**Figure 1:**
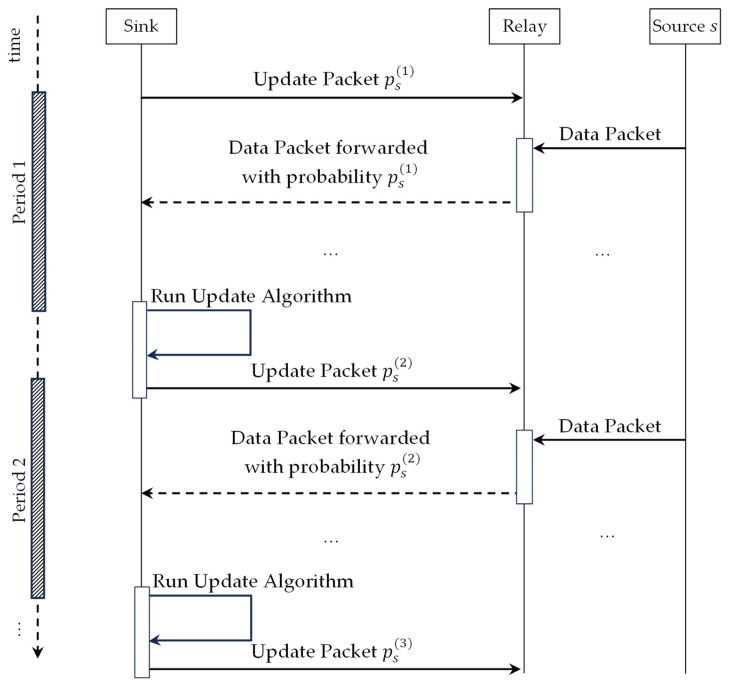
UML sequence diagram of ALSR protocol operation.

The operation of the proposed ALSR protocol divides the time into equal intervals called update periods. At the beginning of each update period, the sink node broadcasts a signed update packet using simple flooding. The update packet for the nth period contains a list of pairs $\left(s,p_{s}^{\left(n\right)}\right)$ for each source node, s. Upon receiving the update packet from the sink, relay nodes use the forwarding probabilities received to tune the DPR protocol operation for delivering packets from sources to the sink. At the end of each period, the sink estimates the current threat level corresponding to each source node and adapts the forwarding probabilities accordingly. The protocol operation is illustrated by a UML sequence diagram in Figure 1.

**Figure 2:**
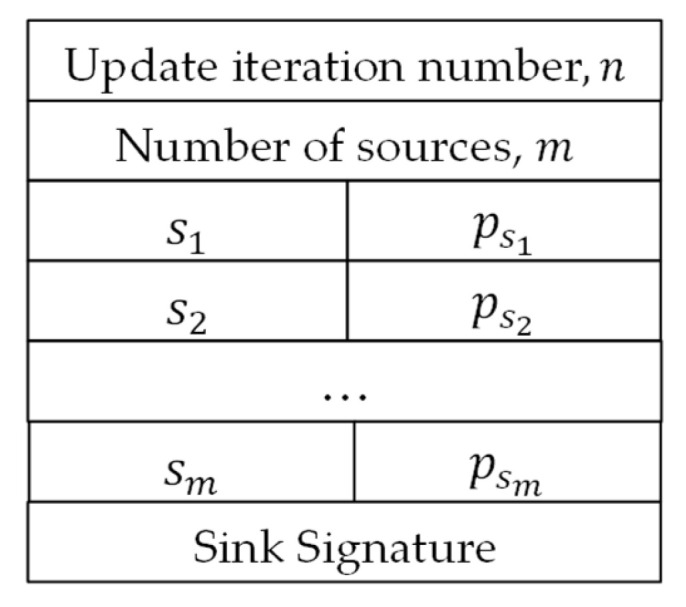
Update packet format.

**Figure 3:**
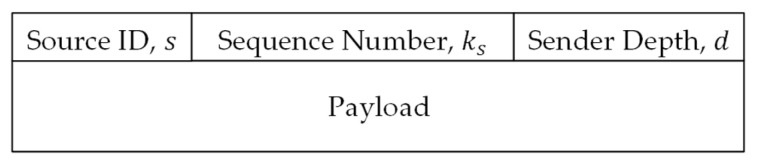
Data packet format.

Two types of packets are employed by the protocol. The first type is “Update Packets”, which are generated by the sink and broadcast to all nodes to tune the per-source forwarding probability. The other type is “Data Packets”, which are generated by source nodes and forwarded using probabilistic depth-based routing to be received by the sink. The formats of the two types of packets are illustrated in Figure 2 and Figure 3, respectively. Figure 2 depicts the format of update packets sent periodically by the sink. The update packet starts with the iteration number field, n, which serves as a sequence number of the update packet. The next field is the number of sources, m, which identifies the number of update pairs included in the update packet. Consequently, the packet contains m pairs of node Id and probability, $\left(s_{i},\;p_{s_{i}}\right)$. Finally, the update packet is protected against tampering using a digital signature generated by the sink node. Figure 3 depicts the format of a data packet, which is composed of a header and a payload. The header contains the Id of the source node, s, a source-unique sequence number, $k_{s}$, and the depth, d, of the packet latest sender.

In the following subsection, we present algorithms for calculating updated forwarding probabilities at the end of each update period.

### 3.3. ALSR Probability Update Algorithms

#### 3.3.1. Lightly Loaded ALSR (ALSR-LL)

The sink node first calculates a threat probability $t_{s}^{\left(n\right)}\in \left[0,1\right]$ for each source node s∈S using the following heuristic approach based on (a) the previous threat estimate $t_{s}^{\left(n-1\right)}$, and (b) the delivery ratio of data coming from node s during this period, denoted $r_{S}^{\left(n\right)}$, as follows

(2) $$t_{s}^{\left(n\right)}=\min\left\{1,\left(K(t_{s}^{\left(n-1\right)}-r_{s}^{\left(n\right)}+1)\right)\right\}$$

The corresponding forwarding probability is given by the formula.

(3) $$p_{S}^{\left(n\right)}=1-\sqrt[{D}]{1-t_{S}^{\left(n\right)}}$$

where D denotes the minimum node degree, i.e., the minimum number of neighbor nodes within the node’s communication range. To justify Equation (3), let us calculate the probability of a successful sinkhole attack corresponding to an unqualified forwarding probability, $p_{s}^{\left(n\right)}$. If the attacker, denoted *A*, has $N_{A}$ neighbors within its range, the probability of a successful attack is equal to the probability of dropping a packet received from s by all A’s neighbors, which is at most ${\left(1-p_{S}^{\left(n\right)}\right)}^{N_{A}}$. Substituting $p_{s}^{\left(n\right)}$ from Equation (3), the probability of a successful attack, $P_{sinkhole}$ is

$$P_{sinkhole}={\left(1-\left(1-\sqrt[{D}]{1-t_{S}^{\left(n\right)}}\right)\right)}^{N_{A}}={\left(1-t_{s}^{\left(n\right)}\right)}^{N_{A}/D}.$$

Since D is the minimum node degree, then $N_{A}⁄D\geq 1$.

$$∴P_{sinkhole}\leq 1-t_{s}^{\left(n\right)}$$

Omitting losses due to the network’s intrinsic limitations, the actual delivery ratio, $r_{s}^{\left(n\right)}$, can be calculated as

$$r_{S}^{\left(n\right)}=1-P_{sinkhole}\geq 1-(1-t_{S}^{\left(n\right)})=t_{S}^{\left(n\right)}\;\;∴r_{s}^{\left(n\right)}\geq t_{s}^{\left(n\right)}$$

For ALSR-LL to successfully converge while the attack is active, we would like the steady state actual delivery ratio $r_{s}^{*}=K$. Equation (2) has a fixed point when $r_{s}^{*}=t_{s}^{*}=K$, which is the desired delivery ratio.

#### 3.3.2. Fast-Recovery ALSR (ALSR-FR)

The threat probability is updated as follows. If the delivery ratio, $r_{s}$, is below the desired delivery ratio, K, by more than a tolerance threshold ϵ, i.e., $r_{s}<K-\epsilon$, the forwarding probability is increased by

(4) $$p_{s}^{\left(n\right)}=p_{s}^{\left(n-1\right)}+\eta_{1}\left(K-r_{s}\right)(1-p_{s}^{\left(n-1\right)})$$

where $\eta_{1}$ is a tunable parameter. On the other hand, if the delivery ratio is above the desired delivery ratio by more than the tolerance threshold, i.e., $r_{s}>K+\epsilon$, the forwarding is decreased.

(5) $$p_{s}^{\left(n\right)}=\max\left\{0,\left(p_{s}^{\left(n-1\right)}-\eta_{2}\left(r_{s}-K\right)\right)\right\}$$

where $\eta_{2}$ is another tunable parameter. Otherwise, if the delivery ratio is within the desirable range, i.e., $\left|r_{s}-K\right|\leq \epsilon$, the forwarding probability remains as it is, i.e.,

(6) $$p_{s}^{\left(n\right)}=p_{s}^{\left(n-1\right)}$$

The three rules can be combined in the following formula.

$$p_{s}^{\left(n\right)}=\left\{\begin{array}{ll} p_{s}^{\left(n-1\right)}+\eta_{1}\Delta \left(1-p_{s}^{\left(n-1\right)}\right), & \Delta >\epsilon \\ \max\left\{0,\;\left(p_{s}^{\left(n-1\right)}+\eta_{2}\Delta \right)\right\}, & \Delta <-\epsilon \\ p_{s}^{\left(n-1\right)}, & otherwise \end{array}\right.$$

where $\Delta =r_{s}-K$ The two parameters $\eta_{1}$ and $\eta_{2}$ can be tuned to adjust the convergence speed, and consequently, the stability of the system.

When the desired delivery ratio is small, a high convergence speed causes the algorithm to overshoot, reaching a forwarding probability greater than the optimal value. On the other hand, when the desired delivery ratio is large, a low convergence speed requires many iterations to reach the optimal forwarding probability. Through experiments, we propose the following empirical formula for calculating a suitable convergence speed.

(7) $$\eta_{1}=\eta_{2}=K^{2}+0.1$$

#### 3.3.3. Polynomial-Fitting ALSR (ALSR-PF)

Two factors affect the relationship between the forwarding probability and the delivery ratio: (1) network congestion, and (2) resilience to depth-spoofing attack. As forwarding probability increases, more packets are forwarded by unqualified forwarders, causing an increase in network traffic, and hence, higher levels of interference and collisions. Thus, higher forwarding probabilities can potentially reduce successful delivery ratios. On the other hand, when the network is under attack, a higher forwarding probability increases the probability of circumventing the attacker and thus increases the delivery ratio. The optimal forwarding probability is the point that maximizes the utility function, i.e., the delivery ratio, by striking a balance between the effect of higher traffic load and the effect of the depth-spoofing attack, if any.

The utility function is modeled as a polynomial with one peak which is obtained and used for optimizing the forwarding probability using the following distributed algorithm.

**ALSR-PF Sink Operation Algorithm:**

1. Initialize forwarding probability $p_{s}^{\left(1\right)}=0$, for all sources s∈S
2. At the end of the first period, set $p_{s}^{\left(2\right)}=0.5$, for all s∈S such that $r_{s}^{\left(1\right)}<K$.
3. At the end of nth update period,
  - for each s∈S:
    1. Estimate the delivery ratio $r_{s}^{\left(n\right)}$ as the ratio of the number of unique packets received from source s during the nth update period to the expected number of packets generated.
    2. Add a new point $\left(p_{s}^{\left(n\right)},r_{s}^{\left(n\right)}\right)$ to the temporal data series ${\left\langle \left(p_{S}^{\left(i\right)},r_{S}^{\left(i\right)}\right)\right\rangle }_{i=1}^{n}$ observed over the past n−1 periods.
    3. Calculate $r_{s}=f\left(p_{s}\right)$ as a least-squares fourth-degree polynomial curve fitting of the series ${\left\langle \left(p_{S}^{\left(i\right)},r_{S}^{\left(i\right)}\right)\right\rangle }_{i=1}^{n}$.
    4. Let P be the set of real roots of the equation f(p)=K such that p∈[0,1].
    5. Let $p_{s}^{\left(n+1\right)}=\left\{\begin{array}{ll} \underset{p\in P}{\min}\;p, & P\neq \phi \\ \underset{p\in \left[0,1\right]}{\mathrm{argmax}}\;f\left(p\right), & otherwise \end{array}\right.$
  - Generate, sign, and broadcast an update packet using flooding.

**ALSR-PF Relay Operation Algorithm**

1. Upon receiving an update packet, a relay node verifies the sink signature, then updates its local forwarding probability table with the new pairs $\left(id_{S},p_{S}\right)$
2. When a relay node, R, receives a packet originating from source S, with sequence number $k_{s}$, the packet is held in the local buffer for the holding time, which is calculated using the classical DBR mechanism.
3. If the relay node, R, overhears a retransmission of the same packet, i.e., originating from S with sequence number $k_{s}$:
  - If the forwarding probability $p_{S}=0$ in the local forwarding table, the buffered packet is dropped immediately.
  - Otherwise, the packet is marked for dropping but remains in the buffer.
4. If a packet holding time expires:
  - If the packet has been marked for dropping, it is forwarded with probability $p_{S}$.
  - Otherwise, i.e., if the packet was not marked for dropping, it is forwarded unconditionally.

The utility function is modeled as a fourth-degree polynomial because as the degree of the polynomial fitting is increased above the fourth degree, the noise in measurements causes anomalies in the resulting function. Our experiment results show that the approximation function does not behave better when the polynomial degree is increased beyond that.

## 4. Evaluation Methodology

### 4.1. Metrics for Evaluating Update Algorithms

The performance of any secure routing protocol is firstly measured by its delivery ratio in the presence of attacks. Since the proposed protocol is adaptive, the delivery ratio drops at the onset of the attack. But once detected, the forwarding probability update algorithm kicks in to readjust the forwarding probability to mitigate the attack and improve delivery ratio. The extra energy required to defend the network against the attack is another important parameter that must be considered when evaluating secure routing algorithms. To evaluate the effectiveness of different update algorithms, we propose the following four metrics.

#### 4.1.1. Delivery Ratio Under Attack

The first metric is the probability of achieving the desired delivery ratio when the network is under attack, provided that the network can achieve it under normal conditions. To measure this probability, we apply each evaluated algorithm on N networks and observe the number of networks, M, for which the algorithm in question achieves the desired delivery ratio within 30 iterations while under depth-spoofing attack. The success probability, M/N, indicates the efficiency of the algorithm in question.

#### 4.1.2. Energy Cost at Steady State

Since sensor networks often have constrained energy resources, another important metric for evaluating a secure routing protocol is delivery efficiency. As defined in [4], energy cost is the total energy spent by the network to deliver one packet. If an algorithm successfully achieves the desired delivery ratio for a certain network, the number of packets transmitted per single delivered packet indicates the delivery efficiency at steady state. We consider only data packet transmission energy because idle receiving energy, processing energy, and control packet overhead are all marginal.

#### 4.1.3. Convergence Time

The faster the response of an update algorithm to the attack, the more effectively the algorithm can be considered. To estimate the response speed, we observe the number of iterations required to converge with the desired delivery ratio. The observation is repeated for N networks and the average convergence time for an update algorithm is considered a metric for the efficiency of the algorithm. A network for which an algorithm does not converge within a reasonable number of update iterations is considered divergent.

#### 4.1.4. Routing Resilience

This metric aims to measure the ability of the protocol to avoid getting packets stuck at nodes with local minimum depth by allowing suboptimal forwarders to participate in the routing. We observe N networks and calculate the percentage of the networks in which the protocol successfully delivered packets to the sink.

### 4.2. Simulation Environment

For evaluating the performance of the proposed protocol, we use the Underwater Acoustic Network Simulator (UANSim) [17]. We adopt the simulation settings detailed in [3] and summarized in Table 2. Namely, each network is formed by placing nodes randomly in a 500 m × 500 m × 250 m volume, ensuring a connected topology with the number of nodes and node degrees corresponding to intended network density: sparse networks have 47 nodes with a minimum degree of 5, medium density networks have 75 nodes with a minimum degree of 8, and dense networks have 150 nodes with a minimum degree of 15. Nodes remain stationary during the simulation. A channel model with spherical spreading, additive noise, and absorption was implemented, aligning with Ref. [4]. Path loss, noise, and bit error rates were calculated using physical layer models assuming BPSK modulation. The ALOHA MAC protocol was used due to high propagation delays in underwater environments, which renders synchronized protocols impractical. Our experiments were designed with a single source node generating 100 packets with a 50-byte payload at 10 bytes/s. The depth-spoofing attack model assumes that a single malicious node is placed right next to the source node to maximize its ability to launch a blackhole attack.

### 4.3. Simulation Scenarios

Each sample network is simulated in five scenarios.

1. The first and second scenarios utilize the non-adaptive DPR protocol with the forwarding probability set to the optimal value proposed in [4], corresponding to the network density, as shown in Table 3.
  - In the first scenario, the network is operating under normal conditions.
  - In the second scenario, the network is under the depth-spoofing attack.
2. The remaining scenarios simulate networks that use the proposed ALSR-LL, ALSR-FR, and ALSR-PF, respectively, while the networks are under depth-spoofing attacks. For each of the proposed adaptive protocols, the simulation is run for the duration of 30 update intervals.

### 4.4. Behavioral Categorization of Simulated Networks

To demonstrate the different convergence behavior of the proposed update algorithms, ALSR-LL, ALSR-FR, and ALSR-PF, we divide the simulated networks into four categories.

For the first category of networks, all three update algorithms converge to the desired delivery ratio of K=0.8. For the second and third categories of networks, ALSR-LL diverges, whereas both ALSR-FR and ALSR-PF converge to the desired delivery ratio. In the second category, ALSR-LL fails to deliver any packets at steady state, whereas in the third category, ALSR-LL continues to oscillate around the desired delivery ratio, leading to intervals in which the delivery ratio is well below the desired level. For the fourth category, both ALSR-LL and ALSR-FR fail to deliver any packets at steady state, whereas ALSR-PF converges with the desired delivery ratio. The convergence behavior of the proposed algorithms with different categories of networks are summarized in Table 4.

**Figure 4:**
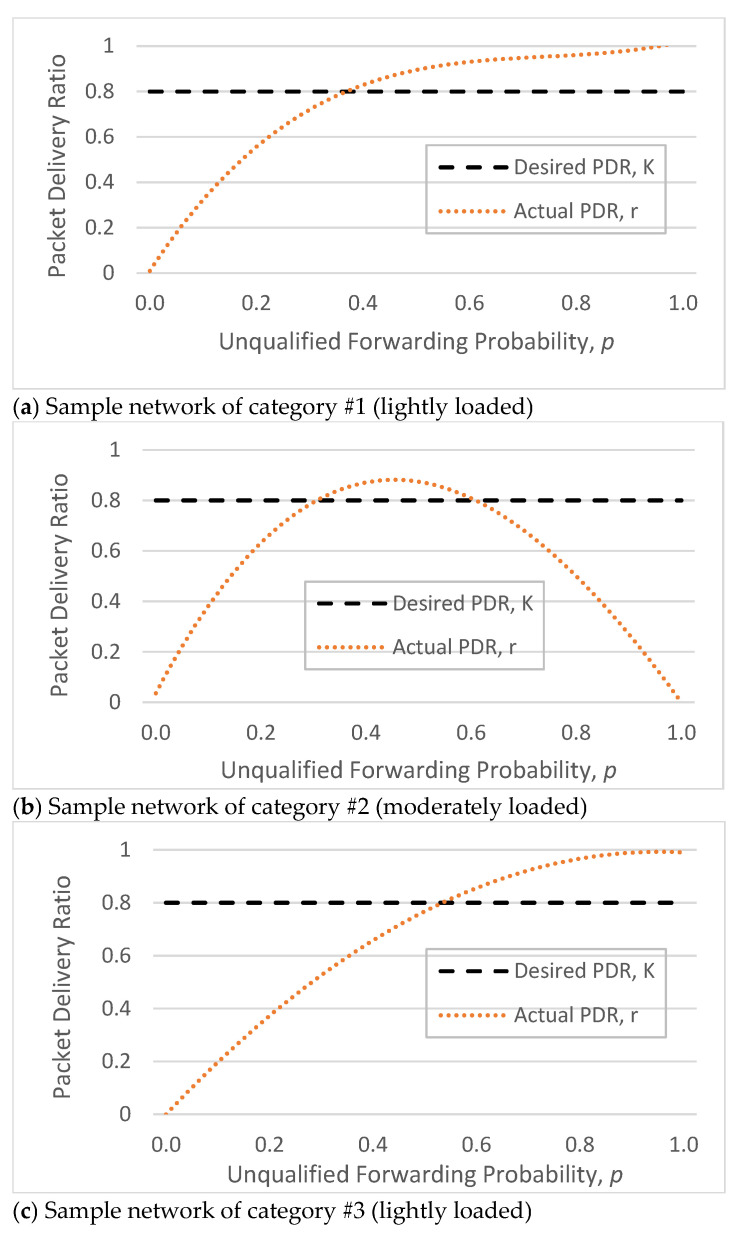

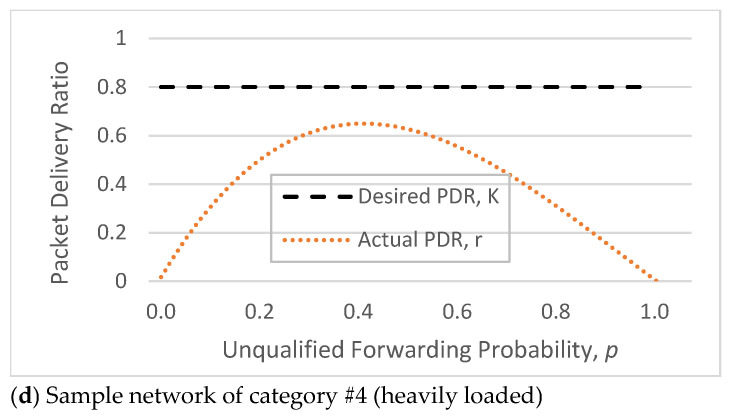
Effect of forwarding probability on DPR delivery ratio in sample networks.

The characteristic curves shown in Figure 4 illustrate the relationship between the DPR forwarding probability parameter and the resulting packet delivery ratio for a representative sample network from each category.

## 5. Results and Discussion

**Figure 5:**
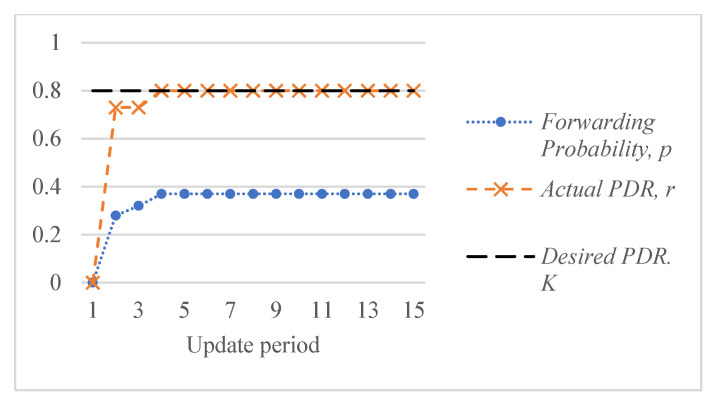
Convergent behavior of ALSR-LL for a sample lightly loaded Network #1 achieving the desired delivery ratio, K=0.8, in three iterations.

**Figure 6:**
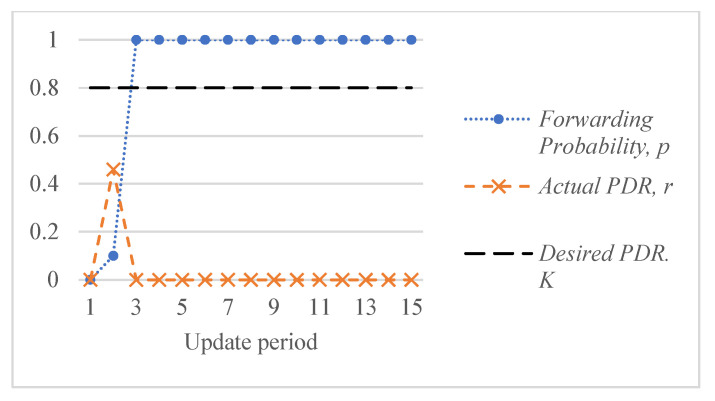
Behavior of ALSR-LL for a sample highly loaded Network #2. The desired delivery ratio K=0.8 cannot be achieved and increasing the forwarding probability beyond 0.62 reduces the delivery ratio.

**Figure 7:**
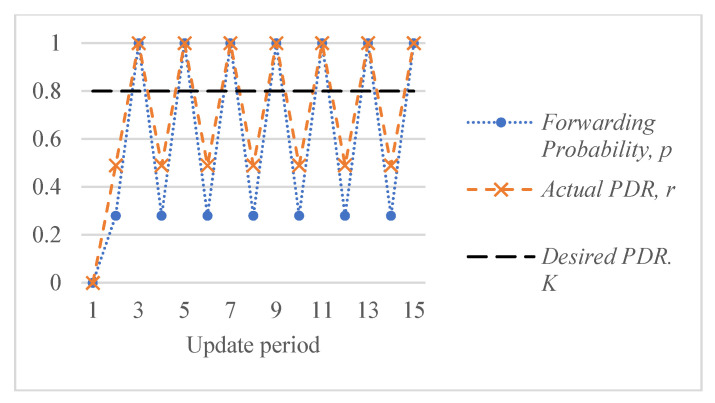
Potential oscillatory behavior of ALSR-LL for a sample Network #3 with a desired delivery ratio K=0.8.

**Figure 8:**
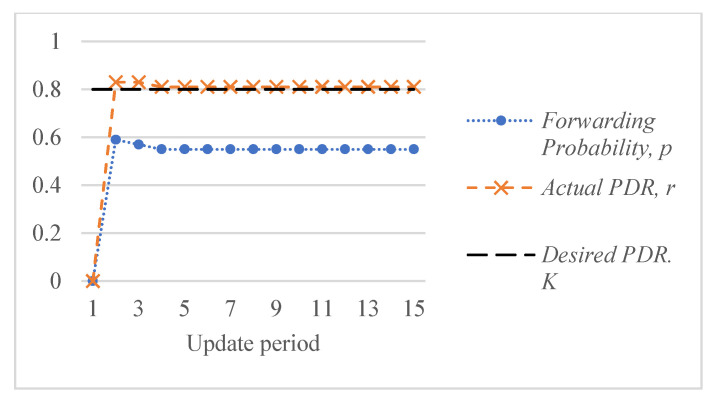
ALSR-FR resolves the oscillatory behavior exhibited by ALSR-LL for sample Network #3 and achieves the desired delivery ratio *K* = 0.8 at forwarding probability *p* = 0.55.

Figure 5 shows an example of the response of the ALSR-LL algorithm to the depth-spoofing attack. The response caused by ALSR-LL to a sudden drop in delivery ratio is a fast rise in the corresponding forwarding probability. This response guarantees a fast mitigation of the black-hole attack. However, if the new delivery ratio after threat mitigation is above the desired ratio, K, the proposed algorithm reduces the forwarding probability to minimize the overhead. In some cases, such as those shown in Figure 5, the forwarding probability converges to the minimum value that achieves the delivery ratio requirement with minimal overhead cost. However, ALSR-LL has some limitations. One limitation of ALSR-LL occurs when the network is moderately loaded such that when the forwarding probability is $p_{s}=1$, severe contention occurs, and the delivery ratio drops below the desired level. An example of such a case is Network #2, shown in Figure 4b. When the forwarding probability exceeds 0.65, the delivery ratio drops below the desired K=0.8 monotonically. Once ALSR-LL increases the forwarding probability beyond this point, ALSR-LL reacts to the declining delivery ratio by further increasing the forwarding probability, which further decreases the delivery ratio. ALSR-LL eventually becomes stuck at the maximum forwarding probability $p_{S}=1$, which is equivalent to flooding. Figure 6 illustrates this limitation of ALSR-LL when handling an attack on this network, leading the network to fail to deliver any packets successfully. Another limitation of ALSR-LL can be seen in some lightly loaded networks, such as Network #3, in which ALSR-LL might oscillate and never reach steady state, as shown in Figure 7. When ALSR-LL raises the forwarding probability beyond what is necessary, the delivery ratio exceeds the desired level. Subsequently, ALSR-LL reduces the forwarding probability to reduce the energy wasted in unnecessary forwarding. The decrease, however, causes the delivery ratio to fall below the desired level. This pattern may continue to occur, causing oscillatory behavior like the one shown in Figure 7. To avoid prolonged instability, the ALSR-FR algorithm controls the convergence speed using the $\eta_{1}$ and $\eta_{2}$ parameters. Figure 8 demonstrates how ALSR-FR resolves the oscillatory behavior exhibited by ALSR-LL when it first overestimates the forwarding probability required to achieve the desired delivery ratio. ALSR-FR slowly reduces the forwarding probability to reduce excessive forwarding while approaching the desired delivery ratio. 

**Figure 9:**
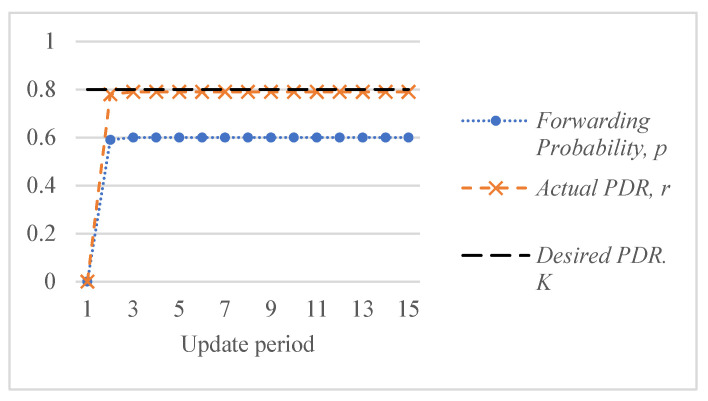
ALSR-FR resolves the problem with overloaded Network #2 and achieves the desired delivery ratio *K* = 0.8 at *p* = 0.6.

**Figure 10:**
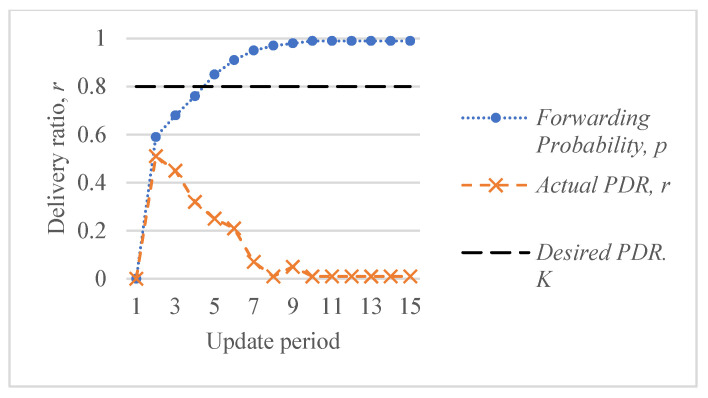
ALSR-FR fails to achieve the desirable delivery ratio, *K* = 0.8, for the highly loaded Network #4.

Moreover, ALSR-FR resolves the first limitation of ALSR-LL in moderately loaded networks. As shown in Figure 9, ALSR-FR successfully achieves the desired delivery ratio in sample network #2 by avoiding the excessive growth of forwarding probability. Although ALSR-FR resolves oscillatory behavior, it may still fail to handle highly loaded networks properly. The behavior of ALSR-FR for Network #4, shown in Figure 10, demonstrates this limitation. When this network is under attack, ALSR-FR first sets the forwarding probability to 0.6. However, the achieved delivery ratio is just 0.51, which is less than the desired K=0.8. When ALSR-FR attempts to increase the forwarding probability to 0.68, the interference increases, and the delivery ratio drops to 45%. As long as the observed delivery ratio is less than desired, ALSR-FR will always attempt to increase the forwarding probability. Initially during the transient period, ALSR-FR behaves slightly better than ALSR-LL but eventually causes the network to fail to deliver packets after a few iterations.

**Figure 11:**
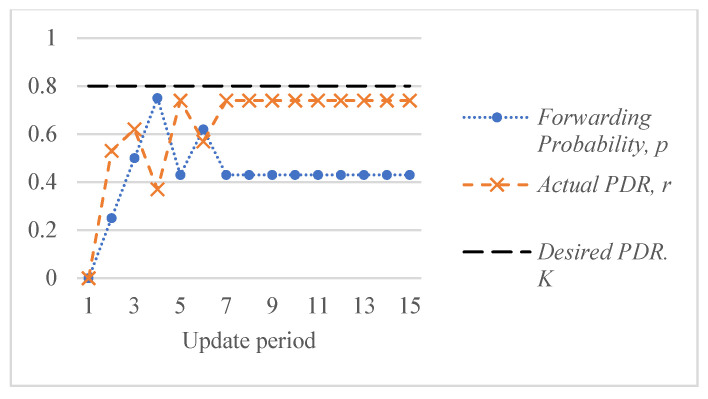
How ALSR-PF handles Network #4 avoiding an excessive increase in forwarding probability.

As evident from Figure 10, when the utility function being maximized is monotonic, i.e., when the network is lightly loaded, both ALSR-FR and ALSR-PF algorithms will achieve the desired delivery ratio. However, if the utility function is nonmonotonic, i.e., when the network becomes congested as the forwarding probability increases, ALSR-FR may fail to achieve the desired delivery ratio. The ALSR-PF algorithm keeps track of past (forwarding probability, delivery ratio) pairs and uses them to approximately calculate the steady state operating point that satisfies the desired delivery ratio. As shown in Figure 11, ALSR-PF manages to handle the depth-spoofing attack against the heavily loaded Network #4 to achieve the maximum possible delivery ratio, which is slightly lower than the desired level.

To compare the evaluation metrics of the proposed update algorithms, namely, the packet delivery ratio, the energy cost at steady state, the convergence speed, and the routing resilience, we simulate 300 networks divided among the categories defined in Table 4, and for different cases of the desired delivery ratio, K. For each network simulation, we observe (1) whether the delivery ratio converged to the desired value, (2) the number of iterations the update algorithm takes to converge, (3) the total number of data packet transmissions throughout the network, and (4) the number of packets successfully delivered to the sink. The results for K = 0.5, K = 0.8, and K = 1.0 are summarized in Table 5, Table 6, and Table 7, respectively. The performance metrics for traditional DBR under normal conditions are provided for reference. Results show that ALSR-PF achieves the best delivery ratio and energy efficiency for all values of K. Moreover, the ALSR-PF algorithm achieves the best energy efficiency in steady state. On the other hand, the ALSR-LL and ALSR-FR algorithms achieve better convergence speed by reaching the desired delivery ratio within as low as one update iteration on average iterations when K=1, which is several times faster than the convergence speed of the ALSR-PF algorithm. Moreover, ALSR-PF achieves the best routing resilience by succeeding in delivering packets under attack in 99% of the simulated networks. It is worth noting that DBR protocol without attack achieves only 95% routing resilience, which means that ALSR-PF can be helpful in avoiding routing dead ends that hinder classical greedy DBR protocol from delivering packets.

Due to the approximation nature of the proposed update algorithms, they may partially achieve the desired delivery ratio under attack, K. Since achieving a delivery ratio greater than the desired ratio is not considered an error, we define a one-sided RMSE metric which only considers underperforming as an error, as follows.

$$OSRMSE=\sqrt{\sum \delta_{K}\left(r\right)\cdot {\left(r-K\right)}^{2}},$$

where r is the delivery ratio at steady state, K is the desired delivery ratio, and $\delta_{K}\left(r\right)$ is an error indicator, which takes the value 1 if r<K, and 0 otherwise. The results of evaluating OSRMSE for each of the three update algorithms has been included in Table 5, Table 6 and Table 7.

To gain more insight into the error behavior of the update algorithms, the distribution of the actual delivery ratio is reported in Table 8. The simulation is repeated with varying desired delivery ratios, K = 0.5, 0.8, or 1.0. For each value of *K*, we vary the tolerance between 0.01 and 0.2 and count the percentage of networks in which the actual delivery ratio is at least K−tolerance. The results highlighted in bold font show that for tolerance ≥0.05, the ALSR-FR and ALSR-PF algorithms succeed in at least 80% of the simulated networks. The behavior of the ALSR-LL algorithm is acceptable at K=1.0, but it is unreliable for lower values of K. Therefore, it is recommended to set K=1.0 if ALSR-LL is applied.

## 6. Conclusions and Future Work

In this paper, we presented a comprehensive framework for adaptive forwarding probability tuning in underwater acoustic sensor networks under depth-spoofing attacks, with three specialized update algorithms: Lightly Loaded ALSR (ALSR-LL), Fast-Recovery ALSR (ALSR-FR), and Polynomial-Fitting ALSR (ALSR-PF). Through rigorous simulation in the Underwater Acoustic Network Simulator (UANSim) across four network density categories (sparse, medium, dense), we demonstrate that ALSR-PF achieves the highest routing resilience and delivery efficiency for real-world deployment. Specifically, ALSR-PF consistently attains a high delivery ratio within a limited tolerance (≤20%) from the desired delivery ratio, with a high probability (≥96%) in all simulated network conditions. ALSR resolves oscillatory instability, avoiding catastrophic failure in congested networks, and maximizing delivery ratios in highly loaded scenarios, whereas ALSR-LL and ALSR-FR suffer from limitations. ALSR-LL provides fast mitigation for lightly loaded networks, but it suffers from oscillatory divergence or complete delivery failure in highly loaded networks. ALSR-FR provides the fastest convergence while mitigating oscillations, but it fails to achieve the desired delivery ratio in some highly congested networks. Distinctly, ALSR-PF’s polynomial curve-fitting approach dynamically balances traffic load and attack resilience by identifying the optimal forwarding probability that maximizes the utility function, i.e., the delivery ratio.

The work provided in this paper paves the way for several future directions. Although the performance of ALSR-PF is encouraging, we recognize that there is room for improvement beyond its convergence speed and error tolerance. This could be one direction that future work can explore. The design of the ALSR-PF protocol has several desirable features: it can flexibly support different desired delivery ratios, $K_{S}$, for different sources, providing differentiated service based on the priorities of information sources. Exploring scenarios having multiple sources is another future direction. ALSR, in general, is designed to respond to situations where multiple depth-spoofing attackers might exist in the network. Exploring the effect of depth-spoofing attacks coordinated by multiple malicious nodes and evaluating the resilience of ALSR in the face of such attacks is the third direction for future work.

## Figures and Tables

**Table 1:** Summary of localization-free routing protocols.

Protocol	Security Mechanism	Threat Model Addressed	Adaptivity	Key Limitations
DBR	None	None	None	Vulnerable to depth-spoofing
RPR	Cryptographic authentication + sliding depth window	Depth-spoofing	None	High control overhead, longer paths
DBSR	Encrypted packet headers with pre-distributed keys	Depth-spoofing	None	Limited scalability, key compromise risk
DPR	Probabilistic forwarding	Depth-spoofing	None	Energy overhead when no attack

**Table 2:** Parameters for simulated networks.

Parameter	Setting		
Deployment space	500 m × 500 m × 250 m deep		
	Network density		
	Sparse	Medium	Dense
Number of nodes	47	75	150
Node degree	5	8	15
Channel model	Spherical spreading, additive noise		
Communication range	150 m		
Modulation and bit rate	BPSK at 12.5 Kbit/s		
Data generation rate	10 Byte/s		
Packet payload	50 Bytes		
Total data generated	100 packets		
MAC	ALOHA		

**Table 3:** Optimal DPR forwarding probability for each network density.

Network Density	DPR Optimal p
Sparse	0.9
Medium	0.5
Dense	0.3

**Table 4:** Categories of networks based on convergence behavior with each of the proposed algorithms.

Cat.	Proposed Update Algorithms			Network Density	Network Load
	ALSR-LL	ALSR-FR	ALSR-PF		
#1	Success	Success	Success	Sparse	Light
#2	*r* = 0	Success	Success	Dense	High
#3	Oscillates	Success	Success	Sparse	Light
#4	*r* = 0	*r* = 0	Success	Medium	High

**Table 5:** Performance evaluation at steady state for *K* = 0.5.

		DBR Without Attack	Proposed Update Algorithms Under Attack		
			ALSR-LL	ALSR-FR	ALSR-PF
Delivery Ratio	Mean	93%	40.9%	51.3%	52.7%
	OSRMSE	18.7%	15.2%	8.4%	6.1%
Energy Cost		20.1	28.7	27.0	23.3
Convergence Time		N/A	1.0	2.4	2.1
Routing Resilience		0%	99%	99%	99%

**Table 6:** Performance evaluation at steady state for *K* = 0.8.

		DBR Without Attack	Proposed Update Algorithms Under Attack		
			ALSR-LL	ALSR-FR	ALSR-PF
Delivery Ratio	Mean	93%	59.6%	75.8%	78.8%
	OSRMSE	18.7%	32.9%	20.8%	11.2%
Energy Cost		20.1	27.0	26.4	25.5
Convergence Time		N/A	2.7	2.1	5.3
Routing Resilience		0%	93%	95%	99%

**Table 7:** Performance evaluation at steady state for *K* = 1.0.

		DBR Without Attack	Proposed Update Algorithms Under Attack		
			ALSR-LL	ALSR-FR	ALSR-PF
Delivery Ratio	Mean	93%	90%	90%	93%
	OSRMSE	18.7%	14.5%	14.5%	5.9%
Energy Cost		20.1	28.8	29	22
Convergence Time		N/A	1.0	0.9	7.2
Routing Resilience		0%	92%	92%	99%

**Table 8:** Distribution of actual delivery ratio under attack.

Delivery Ratio Tolerance	Desired Delivery Ratio Under Attack								
	K=0.5			K=0.8			K=1.0		
	LL	FR	PF	LL	FR	PF	LL	FR	PF
0.00	10%	38%	67%	30%	63%	60%	63%	63%	36%
0.01	16%	**80%**	**83%**	43%	82%	77%	74%	16%	**80%**
0.05	27%	**93%**	**95%**	49%	**92%**	**92%**	**86%**	27%	**93%**
0.10	48%	**95%**	**97%**	51%	**93%**	**94%**	**89%**	48%	**95%**
0.20	**84%**	**96%**	**98%**	54%	**93%**	**96%**	**90%**	**84%**	**96%**

The results highlighted in bold font indicate success in at least 80% of the simulated networks.

## Data Availability

The original contributions presented in this study are included in the article. Further inquiries can be directed to the corresponding author.
